# Survival benefit of overweight patients undergoing MitraClip® procedure in comparison to normal‐weight patients

**DOI:** 10.1002/clc.23897

**Published:** 2022-09-07

**Authors:** Karsten Keller, Martin Geyer, Lukas Hobohm, Alexander R. Tamm, Felix Kreidel, Tobias F. Ruf, Michaela Hell, Volker H. Schmitt, Kevin Bachmann, Sonja Born, Eberhard Schulz, Thomas Münzel, Ralph S. von Bardeleben

**Affiliations:** ^1^ Department of Cardiology, Cardiology I University Medical Center Mainz of the Johannes Gutenberg‐University Mainz Mainz Germany; ^2^ Center for Thrombosis and Hemostasis, University Medical Center Mainz of the Johannes Gutenberg‐University Mainz Mainz Germany; ^3^ Medical Clinic VII: Department of Sports Medicine University Hospital Heidelberg Heidelberg Germany; ^4^ German Center for Cardiovascular Research (DZHK) Partner Site Rhein Main Mainz Germany

**Keywords:** edge‐to‐edge repair, MitraClip, mitral valve regurgitation, obesity, survival, transcatheter mitral valve repair

## Abstract

**Background:**

The number of MitraClip® implantations increased significantly in recent years. Data regarding the impact of weight class on survival are sparse.

**Hypothesis:**

We hypothesized that weight class influences survival of patients treated with MitraClip® implantation.

**Methods:**

We investigated in‐hospital, 1‐year, 3‐year, and long‐term survival of patients successfully treated with isolated MitraClip® implantation for mitral valve regurgitation (MR) (June 2010–March 2018). Patients were categorized by weight classes, and the impact of weight classes on survival was analyzed.

**Results:**

Of 617 patients (aged 79.2 years; 47.3% females) treated with MitraClip® implantation (June 2010–March 2018), 12 patients were underweight (2.2%), 220 normal weight (40.1%), 237 overweight (43.2%), and 64 obesity class I (11.7%), 12 class II (2.2%), and 4 class III (0.7%). Preprocedural Logistic EuroScore (21.1 points [IQR 14.0–37.1]; 26.0 [18.5–38.5]; 26.0 [18.4–39.9]; 24.8 [16.8–33.8]; 33.0 [25.9–49.2]; 31.6 [13.1–47.6]; *p* = .291) was comparable between groups. Weight class had no impact on in‐hospital death (0.0%; 4.1%; 1.5%; 0.0%; 7.7%; 0.0%; *p* = .189), 1‐year survival (75.0%; 72.0%; 76.9%; 75.0%; 75.0%; 33.3%; *p* = .542), and 3‐year survival (40.0%; 36.8%; 38.2%; 48.6%; 20.0%; 33.3%; *p* = .661). Compared to normal weight, underweight (hazard ratio [HR]: 1.35 [95% confidence interval [CI]: 0.65–2.79], *p* = .419), obesity‐class I (HR: 0.93 [95% CI: 0.65–1.34], *p* = .705), class II (HR: 0.39 [95% CI: 0.12–1.24], *p* = .112), and class III (HR: 1.28 [95% CI: 0.32–5.21], *p* = .726) did not affect long‐term survival. In contrast, overweight was associated with better survival (HR: 1.32 [95% CI: 1.04–1.68], *p* = .023).

**Conclusion:**

Overweight affected the long‐term survival of patients undergoing MitraClip® implantation beneficially compared to normal weight.

## Graphical abstract

Long‐term survival stratified for weight classes illustrated by Hazard plot for patients treated with MitraClip® implantation stratified for weight classes. Association of weight class compared to normal‐weight with long‐term survival (adjusted for Logistic EuroScore). Survival rate at discharge, 1‐year follow‐up, and 3‐year follow‐up stratified for weight classes.

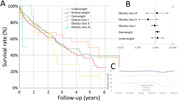

**Abbreviations**
BMI: body mass index
CI: confidence interval
CVD: cardiovascular diseases
DMR: degenerative mitral valve regurgitation
FMR: functional mitral valve regurgitation
HR: hazard ratio
IQR: interquartile range
MR: mitral valve regurgitation
OR: odds ratio
TEER: transcatheter edge‐to‐edge repair (new term of the ACC/AHA guidelines)
WHO: World Health Organization

## 1 INTRODUCTION

Mitral valve regurgitation (MR) is a common heart valve disease in the developed countries^1^, ^2^, ^3^, ^4^ accompanied by substantial morbidity and mortality.^3^, ^5^, ^6^, ^7^, ^8^, ^9^, ^10^, ^11^ The prevalence of MR increases substantially with age^9^, ^10^, ^12^ and patients with MR are frequently referred for surgical or interventional treatment.^2^, ^8^, ^10^, ^12^, ^13^ Transcatheter mitral valve repair (TMVr) using MitraClip® technique or transcatheter edge‐to‐edge repair (TEER) therapy is an established treatment for patients suffering from MR with both primary etiology, who are at high or prohibitive surgical risk, and secondary etiology in a broader range of risk classes and age groups according to current guidelines.^4^, ^6^, ^10^, ^13^, ^14^, ^15^, ^16^ TMVr with MitraClip® implantation has been shown to reduce patients’ symptoms and to improve survival in selected patients.^16^ Thus, the number of TMVr is increasing in Germany and worldwide.^6^, ^10^, ^11^, ^13^, ^14^ Studies indicated that MitraClip® implantations are accompanied by low rates of adverse events.^10^, ^11^, ^12^ However, comorbidities and patient baseline factors potentially influencing long‐term outcomes are still under investigation. Studies have suggested an obesity survival paradox in patients with cardiovascular diseases (CVDs)^17^, ^18^, ^19^ and one recently published study reported an obesity paradox regarding in‐hospital major adverse cardiac events even in TMVR, but failed to confirm an obesity paradox for in‐hospital survival, while data for long‐term follow‐up are entirely missing.^20^ Thus, the objective of the present study was to investigate the impact of nutritional status at the baseline of MitraClip® implantation on patients in short‐term outcomes, and in particular long‐term outcomes.

## 2 PATIENTS AND METHODS

Patients successfully treated for MR (regardless of the underlying pathomechanism, functional MR [FMR] vs. degenerative MR [DMR]) with TEER by MitraClip® implantation in our University Department for Cardiology between June 9, 2010 and March 8, 2018 were included in the present study. Those patients undergoing MitraClip® implantation simultaneously in combination with other forms of TMVr (e.g., direct or indirect mitral valve annuloplasty or chordal reconstruction as COMBO therapy), as well as patients with procedural failure (defined as failure of clip placement due to anatomical reasons or other operator‐reported reasons [e.g., resulting relevant mitral valve stenosis during grasping of the leaflets, leading to the removal of the device and aborting the procedure]), were excluded from this study. All treated patients were aged ≥18 years with moderate to high‐grade or high‐grade MR despite optimal medical treatment and cardiac resynchronization therapy—if indicated—and estimated to be at high risk for valve surgery by an interdisciplinary board (Heart Valve Team).^21^

The individual risk for alternative surgical treatments was calculated with scoring systems (e.g., Logistic EuroScore I, for details and online calculator, see http://www.euroscore.org), and other individual factors such as frailty and comorbidities were taken into account.^21^ All MitraClip® procedures and implantations were guided by fluoroscopy and three‐dimensional transesophageal echocardiography. Procedures were performed under general anesthesia or deep sedation. Long‐term survival or date of death, respectively, were assessed based on entries in our hospital's patients’ records, data from routine follow‐up visits, and an enquiry at the Rhineland‐Palatinate bureau of vital statistics as of March 8, 2018.^21^

### 2.1 Study endpoint

Primary outcome was survival at the following time periods: in‐hospital stay, and 1‐year, 3‐year, and long‐term follow‐up.

### 2.2 Definitions

Based on current guideline recommendations, MR was quantified/categorized into four grades^22^, ^23^: Grade 0 for no or trace MR, Grade I for mild, Grade II for moderate, and Grade III for severe MR. Renal insufficiency was defined as a glomerular filtration rate <60 ml/min·kg. According to the World Health Organization (WHO), underweight was categorized as body mass index (BMI) < 18.5 kg/m^2^, normal‐weight ranges between 18.5 and <25 kg/m^2^, overweight was defined as BMI ≥ 25 and <30 kg/m^2^, obesity class I was defined as BMI between ≥30 and <35kg/m^2^, obesity class II between ≥35 and <40 kg/m^2^, and highest class was obesity class III≥ 40 kg/m^2^. Echocardiographic left‐ and right‐ventricular analysis and quantification were based on transthoracic echocardiography measurements and evaluated in accordance with ASE/EACVI recommendations.^24^

### 2.3 Ethical aspects and study oversight

The study involved only anonymized, retrospective analysis of diagnostic standard data, and thus, individual consent for inclusion was waived according to German law. The study was approved by the local ethics committee on human research and was performed in accordance with the ethical standards laid down in the 1964 Declaration of Helsinki and its later amendments.

### 2.4 Statistics

The included patients were stratified according to the mentioned WHO weight classes and the groups were compared regarding baseline parameters.

Descriptive statistics for the relevant comparisons regarding baseline characteristics of the weight‐class groups were provided with median and interquartile range (IQR), or absolute numbers and corresponding percentages. Continuous variables were compared using the Kruskal–Wallis test and categorical variables with Fisher's exact or *χ* ^2^ test, as appropriate. Univariable logistic regression models were calculated to examine the impact of the different patients’ weight classes in comparison to the normal‐weight group (as the reference group) on in‐hospital mortality, and 1‐year and 3‐year survival. Results were presented as odds ratios (OR) and 95% confidence interval (CI). All of these analyses were performed in a univariable as well as a multivariable manner. Multivariate logistic regression analyses were adjusted for Logistic EuroScore. In addition, Cox regression models were computed to examine the impact of patients’ weight classes in comparison to the normal‐weight group on long‐term survival. These results were presented as hazard ratios (HRs) with 95% CI in a univariable manner as well as in a multivariable manner adjusted for Logistic EuroScore. *p* Values <.05 (two‐sided) were considered to be statistically significant. The software SPSS® (IBM Corp. Released 2016. IBM SPSS Statistics for Windows, Version 25.0: IBM Corp.) was used for computerised analysis.

## 3 RESULTS

Overall, 617 patients (aged 79.2 years in the median; 47.3% females) treated with TMVr using MitraClip® implantations between June 9, 2010 and March 8, 2018, who could be categorized in weight classes, were included in the present study.

### 3.1 Baseline parameters

Patients were categorized as follows: 12 patients were classified as underweight (2.2%), 220 as normal weight (40.1%), 237 as overweight (43.2%), and 64 patients were categorized as obesity class I (11.7%), 12 as obesity class II (2.2%), and 4 as obesity class III (0.7%).

**Figure 1:**
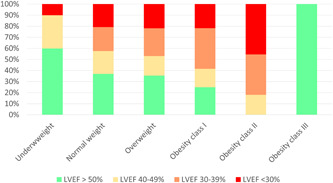
LVEF graduation of the LVEF at baseline of the patients treated with MitraClip® implantation stratified for the different weight classes. LVEF, left ventricular ejection fraction.

**Figure 2:**
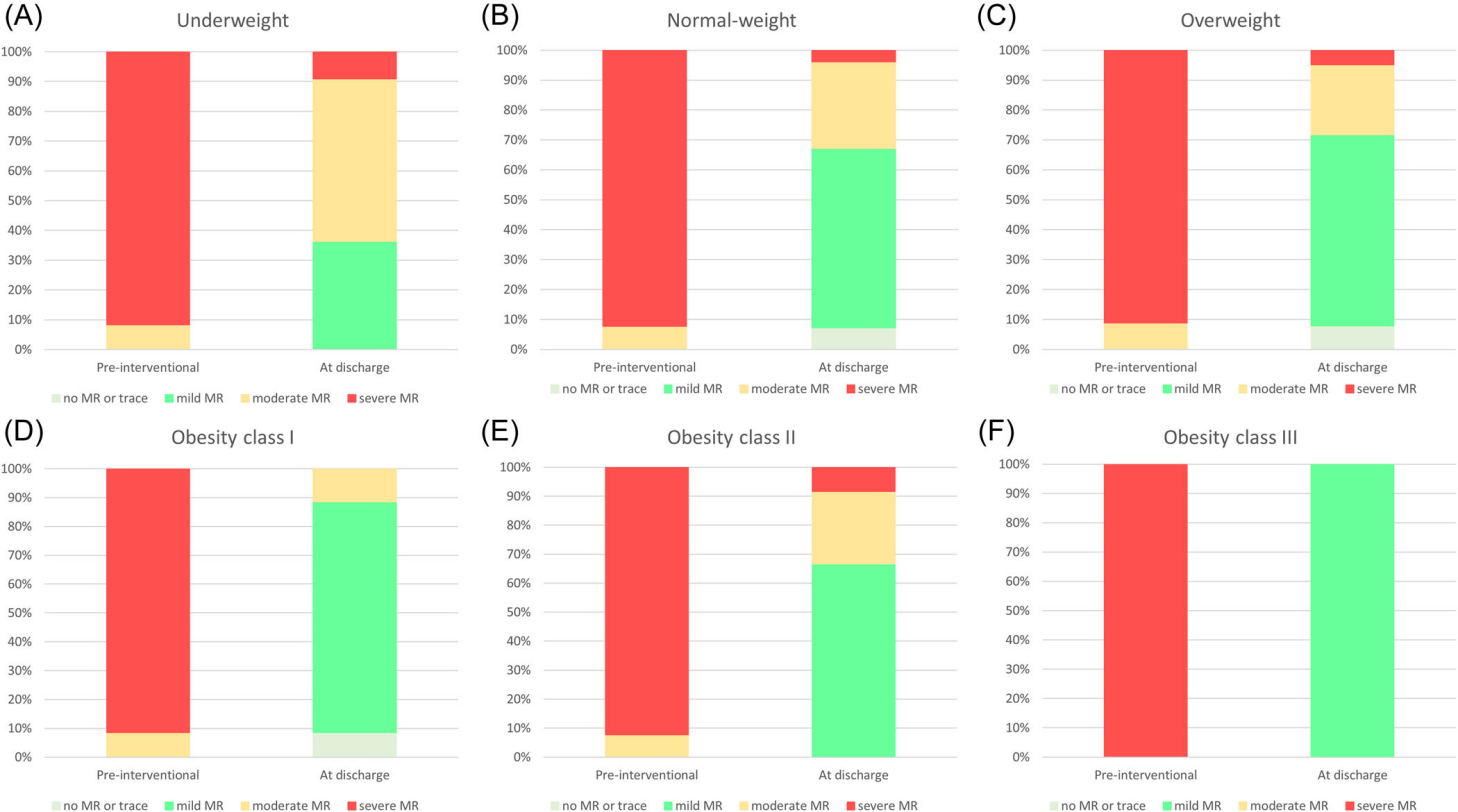
Changes in MR regurgitation severity grades from baseline to discharge in the different weight classes. MR, mitral valve.

Despite higher age in the weight classes underweight and normal weight (underweight: 79.8 [IQR: 72.8–85.8]; normal weight: 80.4 [75.4–84.9]; overweight: 78.7 [74.3–83.5]; obesity class I: 78.7 [72.9–82.9]; obesity class II: 73.3 [70.6–79.0]; obesity class III: 72.5 [70.0–73.1]; *p* < .001), the preprocedural Logistic EuroScore values (21.1 [IQR: 14.0–37.1]; 26.0 [18.5–38.5]; 26.0 [18.4–39.9]; 24.8 [16.8–33.8]; 33.0 [25.9–49.2]; 31.6 [13.1–47.6]; *p* = .291) were comparable between the groups (Table 1). Prevalence of the cardiovascular risk factors arterial hypertension and diabetes mellitus increased in higher weight classes. Additionally, the frequency of renal insufficiency was higher in obese individuals (Table 1). However, the administration of medication for heart failure was similar between the groups. The left ventricular function was more commonly reduced in obese patients of the obesity classes I and II compared to normal‐weight and overweight patients (Table 1 and Figure 1). Figure 2 illustrates the MR grade reduction from pre‐ to postinterventional. Residual MR grades at discharge were comparable between underweight (*p* = .187) and normal weight, overweight (*p* = .528) and normal weight, and class II and normal weight (*p* = .663), as well as class III and normal weight (*p* = .451), whereas patients with obesity class I (*p* = .005) revealed lower residual MR grades at discharge in comparison to normal‐weight patients.

**Table 1:** Baseline characteristics of all patients included in the retrospective analysis stratified for survival status at 1‐year follow‐up

Parameter	Underweight (*n* = 12; 2.2%)	Normal weight (*n* = 220; 40.1%)	Overweight (*n* = 237; 43.2%)	Obesity class I (*n* = 64; 11.7%)	Obesity class II (*n* = 12; 2.2%)	Obesity class III (*n* = 4; 0.7%)	*p* Value
Age at procedure (years)	79.8 (72.8–85.8)	80.4 (75.4–84.9)	78.7 (74.3–83.5)	78.7 (72.9–82.9)	73.3 (70.6–79.0)	72.5 (70.0–73.1)	**<.001**
Age >70 years	10 (83.3%)	227 (93.3%)	238 (87.8%)	56 (80.0%)	10 (76.9%)	4 (80.0%)	.056
Female sex	10 (83.2%)	135 (54.9%)	104 (38.4%)	33 (47.1%)	7 (53.8%)	3 (60.0%)	**.001**
In‐hospital stay (days) after the procedure	5.0 (3.3–6.5)	5.0 (4.0–7.0)	5.0 (4.0–6.0)	5.0 (4.0–7.0)	6.0 (4.5–7.0)	7.0 (5.3–8.8)	.223
NYHA III or IV	10 (83.3%)	190 (86.4%)	211 (89.0%)	63 (98.4%)	12 (100.0%)	3 (75.0%)	.072
Cardiovascular risk factors							
Art. hypertension	8 (66.7%)	200 (81.3%)	244 (90.0%)	60 (85.7%)	13 (100.0%)	5 (100.0%)	**.012**
Diabetes mellitus	0 (0.0%)	40 (16.3%)	85 (31.4%)	35 (50.0%)	9 (69.2%)	3 (60.0%)	**<.001**
Intervention parameters							
FMR	4 (33.3%)	139 (56.5%)	155 (57.2%)	47 (67.1%)	8 (61.5%)	2 (40.0%)	.541
DMR	6 (50.0%)	78 (31.7%)	82 (30.3%)	15 (21.4%)	3 (23.1%)	3 (60.0%)	
Mixed etiology	2 (16.7%)	29 (11.8%)	34 (12.5%)	8 (11.4%)	2 (15.4%)	0 (0.0%)	
Number of implanted clips >1	3 (25.0%)	114 (46.3%)	146 (53.9%)	24 (34.3%)	7 (53.8%)	4 (80.0%)	.126
Logistic EuroScore I (points)	21.1 (14.0–37.1)	26.0 (18.5–38.5)	26.0 (18.4–39.9)	24.8 (16.8–33.8)	33.0 (25.9–49.2)	31.6 (13.1–47.6)	.291
Comorbidities							
COPD	6 (50.0%)	38 (15.4%)	29 (10.7%)	8 (11.4%)	4 (30.8%)	2 (40.0%)	**.001**
Atrial fibrillation	7 (58.3%)	181 (73.6%)	191 (70.5%)	55 (78.6%)	10 (76.9%)	4 (80.0%)	.627
Renal insufficiency	4 (33.3%)	112 (45.5%)	133 (49.3%)	40 (57.1%)	12 (92.3%)	3 (60.0%)	**.014**
Coronary artery disease	5 (41.7%)	154 (62.6%)	172 (63.5%)	53 (76.8%)	8 (61.5%)	4 (80.0%)	.138
Peripheral artery disease	2 (16.7%)	25 (10.2%)	26 (9.6%)	6 (8.6%)	3 (23.1%)	1 (20.0%)	.588
History of stroke	0 (0.0%)	30 (12.2%)	31 (11.4%)	8 (11.4%)	2 (15.4%)	1 (20.0%)	.824
History of aortic valve surgery	1 (8.3%)	15 (6.1%)	24 (8.9%)	5 (7.2%)	2 (15.4%)	1 (20.0%)	.674
Medication							
Diuretics	9 (75.0%)	232 (94.7%)	247 (91.5%)	66 (94.3%)	12 (92.3%)	5 (100.0%)	.137
RAS blockers	9 (75.0%)	200 (81.6%)	223 (82.6%)	59 (84.3%)	11 (84.6%)	4 (80.0%)	.978
Beta‐blockers	7 (58.3%)	206 (84.1%)	224 (83.0%)	55 (78.6%)	10 (76.9%)	4 (80.0%)	.280
Echocardiography							
LVEF (%) pre	52.0 (40.0–55.0)	45.0 (30.0–55.0)	43.0 (30.0–55.0)	35.0 (30.0–50.0)	35.0 (30.0–50.0)	50.0 (50.0–50.0)	**.003**
MR (grade)^a^ preprocedure	3 (3–3)	3 (3–3)	3 (3–3)	3 (3–3)	3 (3–3)	3 (3–3)	.684
MR (grade)^a^ at discharge	2 (1–2)	1 (1–2)	1 (1–2)	1 (1–2)	1 (1–2)	1 (1–1)	**.003**
TAPSE < 1.8 cm	3 (60%)	62 (54.4%)	64 (52.5%)	21 (65.6%)	0 (0%)	1 (100%)	.566
sPAP > 30 mm Hg	8 (100%)	183 (96.3%)	197 (98.5%)	55 (98.2%)	7 (100%)	2 (100%)	.767
Laboratory examinations							
BNP (pg/ml) preprocedure	453.0 (110.3–812.3)	564.0 (276.0–1478.5)	582.5 (275.0–1142.0)	444.0 (219.5–980.5)	888.0 (736.0–1497.0)	357.5 (147.0–888.3)	**.040**
hsTnI (pg/ml) preprocedure	9.2 (4.6–35.5)	19.1 (7.8–46.6)	18.8 (8.2–43.8)	19.2 (6.8–47.3)	32.8 (14.9–73.0)	11.8 (5.1–58.1)	.667

*Note*: Bold values considered to be statistically significant at *p* < .05 (two‐sided).

Abbreviations: BNP, brain natriuretic peptide; COPD, chronic obstructive pulmonary disease; DMR, degenerative mitral valve regurgitation; FMR, functional mitral valve regurgitation; hsTnI, high sensitive troponin I; LVEF, left ventricular ejection fraction; MR, mitral valve regurgitation (^a^classified into four grades: 0 = no/trace, 1 = mild, 2 = moderate or moderate‐severe, 3 = severe); NYHA, New York Heart Association; RAS, renin–angiotensin system; sPAP, systolic pulmonary artery pressure; TAPSE, tricuspid annular plane systolic excursion.

### 3.2 Impact of weight classes on survival

**Figure 3:**
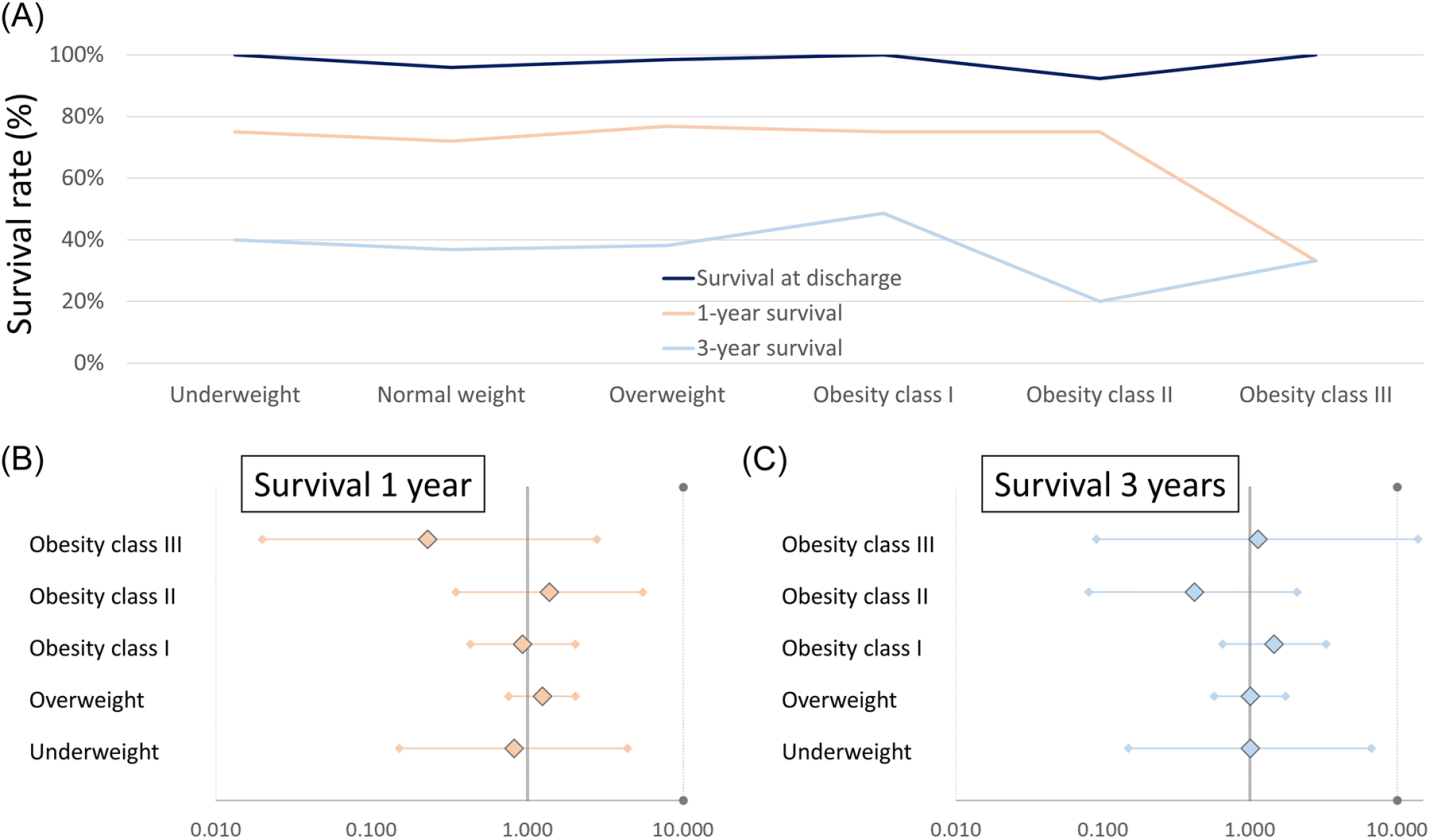
Survival rate at discharge, 1‐year follow‐up, and 3‐year follow‐up stratified for weight classes. (A) Survival rate at discharge (dark blue line), 1‐year follow‐up (orange line), and 3‐year follow‐up (light blue line) stratified for weight classes. (B) Association of weight class compared to normal weight with 1‐year survival (adjusted for Log EuroScore). (C) Association of weight class compared to normal weight with 3‐year survival (adjusted for Log EuroScore).

The weight class had no impact on in‐hospital death (underweight: 0.0%; normal weight: 4.1%; overweight: 1.5%; obesity class I: 0.0%; obesity class II: 7.7%; obesity class III: 0.0%; *p* = .189), 1‐year survival (75.0%; 72.0%; 76.9%; 75.0%; 75.0%; 33.3%; *p* = .542) and 3‐year survival (40.0%; 36.8%; 38.2%; 48.6%; 20.0%; 33.3%; *p* = .661) (Figure 3A).

Underweight (univariable: OR: 1.17 [95% CI: 0.23–5.95], *p* = .854; multivariable: OR: 0.82 [95% CI: 0.15–4.40], *p* = .813), overweight (univariable: OR: 1.30 [95% CI: 0.82–2.05], *p* = .269; multivariable: OR: 1.25 [95% CI: 0.76–2.04], *p* = .386), obesity class I (univariable: OR: 1.17 [95% CI: 0.58–2.35], *p* = .669; multivariable: OR: 0.93 [95% CI: 0.43–2.04], *p* = .864), obesity class II (univariable: OR: 1.17 [95% CI: 0.30–4.47], *p* = .823; multivariable: OR: 1.38 [95% CI: 0.35–5.51], *p* = .649) and obesity class III (univariable: OR: 0.19 [95% CI: 0.02–2.19], *p* = .185; multivariable: OR: 0.23 [95% CI: 0.02–2.78], *p* = .249) were all not associated with 1‐year survival in comparison to the reference group of patients with normal weight (Figure 3B).

Similarly, the 3‐year survival of the patients treated with MitraClip® was not affected by underweight (univariable: OR: 1.14 [95% CI: 0.19–7.08], *p* = .886; multivariable: OR: 1.00 [95% CI: 0.15–6.67], *p* = .997), overweight (univariable: OR: 1.06 [95% CI: 0.65–1.74], *p* = .814; multivariable: OR: 1.00 [95% CI: 0.57–1.75], *p* = .998), obesity class I (univariable: OR: 1.62 [95% CI: 0.78–3.39], *p* = .196; multivariable: OR: 1.45 [95% CI: 0.65–3.27], *p* = .366), obesity class II (univariable: OR: 0.43 [95% CI: 0.09–2.10], *p* = .296; multivariable: OR: 0.42 [95% CI: 0.08–2.09], *p* = .286) or obesity class III (univariable: OR: 0.86 [95% CI: 0.08–9.70], *p* = .901; multivariable: OR: 1.13 [95% CI: 0.09–13.87], *p* = .924) in comparison to individuals with normal weight (Figure 3C).

**Figure 4:**
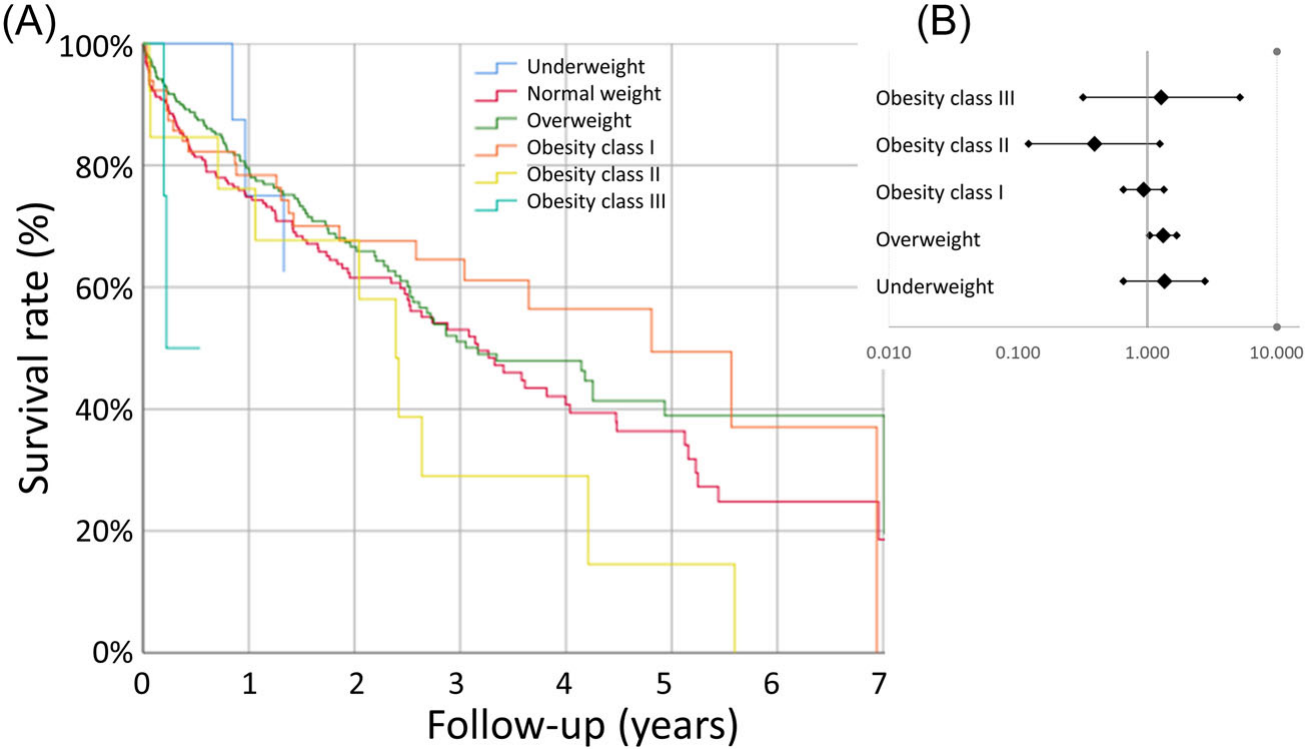
Long‐term survival stratified for weight classes. (A) Hazard plot for patients treated with MitraClip® implantation stratified for weight classes. (B) Association of weight class compared to normal‐weight with long‐term survival (adjusted for Logistic EuroScore).

Regarding long‐term survival, underweight (univariable: HR: 1.68 [95% CI: 0.82–3.44], *p* = .159; multivariable: HR: 1.35 [95% CI: 0.65–2.79], *p* = .419) was not associated with lower long‐term survival in comparison to normal weight. In addition, obesity class I (univariable: HR: 1.01 [95% CI: 0.72–1.43], *p* = .950; multivariable: HR: 0.93 [95% CI: 0.65–1.34], *p* = .705), obesity class II (univariable: HR: 0.37 [95% CI: 0.12–1.17], *p* = .092; multivariable: HR: 0.39 [95% CI: 0.12–1.24], *p* = .112) as well as obesity class III (univariable: HR: 1.46 [95% CI: 0.36–5.94], *p* = .594; multivariable: HR: 1.28 [95% CI: 0.32–5.21], *p* = .726) did also not affect long‐term survival significantly. In contrast, overweight was independently associated with improved survival (univariable: HR: 1.18 [95% CI: 0.94–1.48], *p* = .151; multivariable: HR: 1.32 [95% CI: 1.04–1.68], *p* = .023) in comparison to the reference group with normal weight in the adjusted regression model (Figure 4).

## 4 DISCUSSION

Epidemiological studies and surveys reported an alarming increase in the prevalence of obesity worldwide.^17^, ^20^, ^25^, ^26^ Obesity was associated with poor long‐term outcomes in the healthy general population and was identified as an important risk factor for the development of cardiovascular diseases (CVD), occurrence of CVD events, and increased mortality.^20^, ^25^, ^27^, ^28^ Based on this burden of knowledge, the American joint guidelines of the American Heart Association (AHA) and the American College of Cardiology Foundation of 2011^29^ recommend a weight management and optimization with the primary aim to maintain or achieve a BMI within the normal range between 18.5 and 24.9 kg/m^2^ in particular for patients with CVD.^29^ Despite the unfavorable consequences of obesity on the development of CVD and long‐term outcomes,^25^, ^27^, ^30^ several studies demonstrated that obese patients with CVD,^17^, ^18^, ^19^ such as coronary artery disease as well as myocardial infarction,^17^, ^18^, ^31^, ^32^ pulmonary embolism,^33^ heart failure,^17^, ^34^ and atrial fibrillation^35^ revealed a better prognosis compared to their leaner counterparts.^19^ This phenomenon with a survival discrepancy has been referred to as the term “obesity paradox.”^17^, ^18^, ^19^, ^25^, ^31^, ^36^

Data about the impact of obesity as well as weight classes on mitral valve disease are sparse. One study demonstrated an association between low BMI and the presence of mitral valve prolapse,^37^ whereas the influence of obesity on the development of MR is widely unknown.^20^ While an obesity survival paradox was demonstrated for patients with transcatheter aortic valve implantations,^25^, ^38^, ^39^, ^40^, ^41^ one study failed to confirm an influence of obesity on the outcomes after mitral valve surgery^42^ and one recently published large study was also not able to confirm an obesity survival paradox for the in‐hospital stay of patients treated with MitraClip® implantations.^20^

The results of the present study demonstrated for the first time that overweight affected survival beneficially regarding the long‐term survival of patients treated with TEER, and therefore, the presence of an overweight survival paradox in these patients. The long‐term survival of overweight patients was 1.3‐fold higher than that of the normal‐weight reference group independently of the parameters of the Logistic EuroScore. In contrast, obesity was not associated with better survival.

The reasons underlying the obesity or overweight paradox have still not been fully elucidated and understood.^20^, ^28^, ^43^, ^44^, ^45^, ^46^ Regarding the obesity/overweight paradox, it has to be considered that obesity and overweight might prevent malnutrition and energy wastage.^20^ In this context, it should not be overlooked that patients treated with TEER caused by severe MR with high surgical risk are frequently affected by an end‐stage CVD.^6^, ^13^, ^20^, ^47^ Patients with severe MR often suffer from heart failure (symptoms) and have often a poor prognosis.^47^

Obesity and overweight represent a nutritional reserve, which might particularly become important in older patients with frailty when comorbidities and lower homeostatic reserves coexist.^48^ In addition, overweight and obesity may protect against malnutrition and energy wastage during acute CVD events, surgeries, or interventions based on alterations with respect to the neuroendocrine status that may subsequently have an impact on the modulation of pathologic cardiovascular remodeling.^28^, ^48^, ^49^, ^50^, ^51^ Higher BMI and obesity may protect patients against inflammatory cytokines by an enhanced production of “buffering” lipoproteins.^28^, ^43^

Another hypothesis for the obesity paradox consists of the assumption that normal weight in older individuals with CVD is largely uncommon in Western populations, insinuating that normal weight may reflect the presence of unknown or serious comorbid conditions.^28^, ^52^, ^53^

The apparent discrepancy that overweight affected the long‐term survival of patients treated by TEER for MR beneficially, but obesity did not, might be attributed to the fact that indeed both weight classes (overweight as well as obesity) are related to a nutritional reserve, but obesity might be affiliated to other unfavorable periprocedural and long‐term effects. The higher rate of reduced left ventricular function in obese patients (obesity classes I and II) as well as the higher rate of the postprocedural remaining unfavorable moderate and severe MR in patients with obesity class II might in part be an explanation for a similar long‐term survival of obese patients despite better nutritional reserve of obesity in comparison to normal weight. This finding might outline the specific anatomical challenges and efforts in non‐normal weight patients to achieve optimal MR reduction by TMVr and underlines the importance of a careful patient selection.^54^

### 4.1 Limitations

Some limitations regarding our study merit consideration: First, the design of the study is a monocentric retrospective analysis on an all‐comer sample of patients undergoing interventional edge‐to‐edge repair for MR without any control group. The follow‐up rate was almost complete (96.7%); thus, selection bias can widely be excluded. Second, the potential impact of weight classes on prognosis has to be interpreted with caution since some weight class groups were small.

## 5 FUTURE DIRECTIONS

Although TMVr using MitraClip® technique or TEER therapy is an established treatment for patients suffering from MR with both primary etiology, who are at high or prohibitive surgical risk, and secondary etiology in a broader range of risk classes and age groups according to current guidelines,^4^, ^6^, ^10^, ^13^, ^14^, ^15^, ^16^ identification of patients at higher risk of complications and mortality as well as identification of specific risk factors of poor outcome is of outstanding interest.^54^

## 6 CONCLUSION

Our results demonstrated a long‐term survival benefit for patients undergoing MitraClip® procedure with overweight in comparison to normal weight patients.

## CONFLICTS OF INTEREST

Lukas Hobohm reports having received lecture honoraria from MSD. Felix Kreidel reports having received consultancy and lecture honoraria from Abbott, Cardiac Implants, Edwards Lifesciences. Eberhard Schulz reports lecture honoraria from Edwards Lifesciences and Medtronic. Ralph Stephan von Bardeleben reports having received lecture honoraria from Abbott Structural Heart, Bioventrix, Boehringer Ingelheim, Cardiac Dimensions, Edwards Lifesciences and Philips Healthcare outside the current paper. Unpaid IIT and trial participation as Global or local PI and steering team member to Abbott Structural Heart, Cardiac Dimensions, Edwards Lifesciences, IZKS University of Göttingen, LMU Munich and DZHK Germany. The remaining authors declare no conflict of interest.

## Data Availability

The data that support the findings of this study are available from the corresponding authors upon reasonable request.
